# Pregnancy-Associated Risk Factors of Postpartum Breast Cancer in Korea: A Nationwide Health Insurance Database Study

**DOI:** 10.1371/journal.pone.0168469

**Published:** 2016-12-15

**Authors:** Eun Joo Kang, Jae Hong Seo, Log Young Kim, Geun U. Park, Min-Jeong Oh, Pyoung-Jae Park, Geum Joon Cho

**Affiliations:** 1 Division of Hemato-oncology, Department of Internal Medicine, Korea University Guro Hospital, Korea University College of Medicine, Seoul, Korea; 2 The Health Insurance Review & Assessment Service of Korea, Seoul, Korea; 3 Department of Applied Statistics, Chung-Ang University, Seoul, Korea; 4 Department of Obstetrics and Gynecology, Korea University College of Medicine, Seoul, Korea; 5 Division of Transplant and Vascular Surgery, Department of Surgery, Korea University Guro Hospital, Korea University College of Medicine, Seoul, Korea; Gentofte Hospital, DENMARK

## Abstract

Patients with postpartum breast cancer have been reported to have a poor prognosis. The present study aimed to evaluate the pregnancy-related risk factors of postpartum breast cancer in Korea. We collected patient data from the Korea National Health Insurance (KNHI) Claims Database of the Health Insurance Review and Assessment Service (HIRA) for the 2009–2013 period. We evaluated the pregnancy-related risk factors for postpartum breast cancer in two population groups. For Group 1 (women who had given birth during the 2010–2012 period), data on those who were diagnosed with breast cancer from childbirth to 1-year postpartum were extracted. For Group 2, we extracted the data of women who gave birth in 2010 and traced them until December 31, 2013. In Group 1, 1,384,551 deliveries and 317 postpartum breast cancer patients were recorded in Korea between January 1, 2010, and December 31, 2012. Women aged ≥35 years (Odds Ratio [OR], 2.003; 95% Confidence Interval [CI], 1.567–2.560) and those who gave birth via cesarean delivery (OR, 1.237; 95% CI, 0.986–1.553) were considered to be at a higher risk for breast cancer. Lower risk was noted in primiparous women (OR, 0.737; 95% CI, 0.585–0.928). In Group 2, the data of 457,924 women who gave birth in 2010 were traced until December 31, 2013. Among them, 655 patients were diagnosed with breast cancer, and age ≥35 years and cesarean delivery were associated with an higher risk of breast cancer, whereas primiparous status was associated with a lower risk of breast cancer. In conclusion, older age (≥35 years) and cesarean delivery are significant risk factors for postpartum breast cancer, and primiparous women have a lower risk of developing postpartum breast cancer.

## Introduction

Breast cancer is the most common cancer in women. The risk of breast cancer is closely influenced by various reproductive factors. Epidemiological studies have demonstrated dual opposing effects of pregnancy on the mother’s risk of breast cancer [1,2]. Full-term pregnancy has long-term protective effects against breast cancer in the later part of life; however, the incidence of breast cancer transiently increases after childbirth, and this increased risk is reported to last 10–15 years [3,4]. Although the number of patients diagnosed with breast cancer during pregnancy or postpartum period is small, pregnancy-associated breast cancer (PABC) has been extensively studied. PABC is usually defined as breast cancer diagnosed during pregnancy or within 1–2 years postpartum [5]. The incidence of PABC is estimated to be approximately 1 in 3,000 childbirths [6,7], and the incidence rate has been reported as 11–28.8 per 100,000 deliveries, according to the studies performed in various countries having different definitions for PABC duration [8–10]. For breast cancer during postpartum compared to breast cancer during pregnancy, the incidence of breast cancer diagnosed postpartum is higher than the incidence of breast cancer diagnosed during pregnancy [8,11]. According to the California Cancer Registry, postpartum breast cancer, diagnosed within a 12-month period, was recorded in 669 cases among 4,846,505 obstetric deliveries while 266 cases were recorded during pregnancy between 1991 to 1999 [8]. In another study, a higher number of breast cancer cases were detected, more than expected, during 7–12 months postpartum and second year after childbirth; the ratio of observed number per expected number (O/E ratio) was 1.12 (1.01–1.24) in 7–12 months postpartum and 1.10 (1.03–1.18) in the second year after childbirth, while the number of breast cancer cases detected during pregnancy was lower than expected, with an O/E ratio of 0.35 between 1963 to 2007 [11]. Moreover, increasing incidence rate of PABC has been reported. In a population-based cohort study using data from the Swedish Cancer Registry, the incidence of PABC increased from 16.0 patients in the first calendar period to 37.4 patients in the last calendar period per 100,000 deliveries between 1963 and 2002 [12]. Complex factors might cause the increasing incidence of PABC; however, global trend toward postponed childbearing is thought to be an influential factor because delayed childbirth, such as maternal age greater than 35 at first birth, is associated with increased risk of PABC [1–4].

With improvement in treatment modalities and early detection, the overall survival of patients with breast cancer has improved. However, PABC in young women is still a burden for patients, families, and physicians because it is known to have a poorer prognosis. Several previous studies reported that PABCs are potentially large advanced stage tumors with high histological grades and they tend to have negative hormone receptor status [13–15]. In addition, patients in their pregnancy and postpartum periods easily neglect and misinterpret their symptoms as pregnancy-related, owing to the changes in the context and density of the mammary tissue to dense or hyperplastic during pregnancy and postpartum. Hyperplastic change of mammary tissue makes it difficult to differentiate normal tissue changes from abnormal mass; consequently, this change causes a delay in early detection of breast cancer using self-examination or mammography. Moreover, mothers are reluctant to undergo diagnostic procedures during pregnancy because of their concern for the fetus. These factors also lead to delayed diagnosis, resulting in poor prognosis.

Therefore, identification of predictive or risk factors is necessary for the early detection of PABC in pregnant women and during postpartum period. Since women in their pregnancy and postpartum visit the hospital more frequently than those who are not pregnant, the investigation of PABC-related risk factors, which can be detected during pregnancy or postpartum, would improve the diagnosis of breast cancer in these patients. Therefore, we investigated the pregnancy-associated risk factors of PABC such as obstetric complications, modality of delivery, twin pregnancy, and parity for the early prediction of PABC. However, it is difficult to know whether obstetric factors in patients diagnosed with breast cancer during pregnancy are related to the cause or outcome of cancer. Therefore, using the nationwide database, we focused on investigation of the pregnancy-related risk factors only in patients diagnosed with breast cancer in the postpartum period. We investigated the differences between characteristics of women diagnosed with breast cancer within a year after childbirth and those who were not diagnosed with breast cancer. Then, we conducted investigation on pregnancy-related risk factors in this population. In addition, we traced the data of patients diagnosed with breast cancer after childbirth in 2010 over a maximum time period of 4 years. The number of cases of postpartum breast cancer over time and pregnancy-related risk factors in this population were also investigated.

## Materials and Methods

The study data were collected from the Korea National Health Insurance (KNHI) Claims Database of the Health Insurance Review and Assessment Service (HIRA) for the 2009–2013 period. In Korea, 97% of the population is obligated to enroll in the KNHI program. Healthcare providers are required by the health insurance policies to allow the review of medical costs incurred, by HIRA. The remaining 3% of the population is under the Medical Aid Program. Thus, the HIRA database contains information on all claims for approximately 50 million Koreans, and nearly all of the information concerning the volume of disease can be obtained from this centralized database, with the exception of procedures that are not covered by insurance, such as cosmetic surgery. Many epidemiological analyses have been published using this database. According to the Act on the Protection of Personal Information Maintained by Public Agencies, HIRA prepares the claims data by concealing the individuals’ identities. The database we received included an unidentifiable code representing each individual, together with information on their age, diagnosis, and a list of prescribed procedures.

The diagnosis and procedure codes from the tenth revision of the International Classification of Diseases (ICD-10) were used to identify women who gave birth and those diagnosed with breast cancer during the study period. The current study included women who had given birth during the 2010–2012 period only. The study population was divided into two groups for analysis: Group 1 and Group 2. For Group 1, investigation of different characteristics and obstetric risk factors among women who had given birth during the 2010–2012 period and who had been diagnosed with postpartum breast cancer within 1 year after childbirth was performed. Included women were classified as having postpartum breast cancer if they were diagnosed with breast cancer (ICD-10 code, E11) in the period from the time of delivery to 1-year postpartum, and underwent surgery and/or chemotherapy for breast cancer. Since patients who were diagnosed with breast cancer before delivery could also make medical claim on breast cancer after delivery, we added one additional criterion for the exclusion of patients who were diagnosed with breast cancer before delivery. Therefore, only patients who were diagnosed with breast cancer (ICD-10 code, E11) after delivery and also did not have medial claim for breast cancer before delivery, beginning January 1, 2009 were confirmed as patients who were diagnosed with postpartum breast cancer. For the Group 2, we investigated the incidence of postpartum breast cancer over a time period of maximum 4 years in women who gave birth in 2010. Therefore, the data of women who gave birth in 2010 were traced until December 31, 2013, and they were classified as having postpartum breast cancer if they were diagnosed with breast cancer (ICD-10 code, E11) until December 31, 2013 and underwent surgery and/or chemotherapy for breast cancer. Furthermore, only patients who did not have a medial claim for breast cancer before delivery, beginning January 1, 2009 were confirmed as patients having postpartum breast cancer.

The data collected from the database on women’s characteristics were age, multiple pregnancies (defined as twin or higher-order gestation), number of deliveries, delivery mode (vaginal delivery or cesarean section), complications of pregnancy including gestational diabetes mellitus, and pre-eclampsia.

Student’s t-test was performed to compare the continuous variables between postpartum breast cancer group and non-breast cancer group, while Pearson chi-square test was used to compare categorical variables. To evaluate the risk of breast cancer development after delivery, multivariate logistic regression analysis was performed including the development of breast cancer after delivery as the outcome within the entire study population. All statistical analyses were performed using SPSS software, version 20 (SPSS Inc., Chicago, IL, USA), and a P value <0.05 was considered statistically significant.

## Results

**Fig 1:**
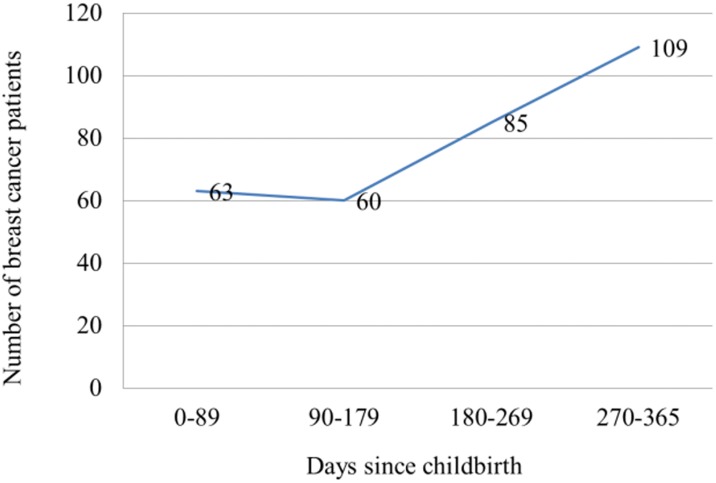
The number of patients diagnosed with postpartum breast cancer (diagnosed within 365 days after childbirth) among mothers who delivered during the 2010–2012 period.

**Fig 2:**
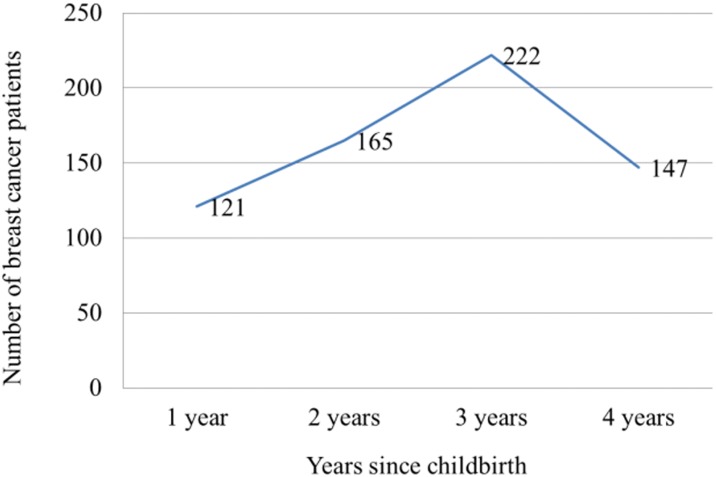
The number of patients diagnosed with breast cancer after delivery among mothers who delivered in 2010.

In Group 1, 1,384,551 deliveries were recorded in Korea between January 1, 2010 and December 31, 2012. Among them, 317 postpartum breast cancer patients who were diagnosed with breast cancer within 365 days after childbirth were recorded. The number of postpartum breast cancer patients was counted for each quarter. The number of patients diagnosed with breast cancer increased with time; 63 patients were diagnosed with breast cancer within 89 days after childbirth, 60 patients within 90–179 days, 85 patients within 180–269 days, and 109 patients within 270–365 days (Fig 1). Table 1 shows the characteristics and numbers of patients with or without breast cancer diagnosis in Group 1. The proportion of old age women (≥35 years) was higher among mothers with breast cancer than those without breast cancer. In addition, the proportion of primiparous women and women who underwent cesarean delivery were higher among mothers with breast cancer than among those without breast cancer. Pre-eclampsia, gestational diabetes, and twin delivery were not associated with breast cancer. Next, we investigated the multivariate-adjusted odd ratios (ORs) for breast cancer. Old age (≥35 years), cesarean delivery, and primiparous status were associated with a risk of breast cancer. Higher risk was noted in women aged ≥35 years (OR, 2.003; 95% confidence interval [CI], 1.567–2.560) and women who underwent cesarean delivery (OR, 1.237; 95% CI 0.986–1.553). Lower risk was noted in primiparous women (OR, 0.737; 95% CI, 0.585–0.928; Table 2). For the analysis of Group 2, we investigated 457,924 mothers who delivered in 2010, and their medical insurance claim records were traced until December 21, 2013. During that period, 655 patients were diagnosed with breast cancer. The number of patients diagnosed with breast cancer increased during the 3 years after delivery. Among mothers who had delivered in 2010, the number of patients who were diagnosed with breast cancer within the first year after delivery was 121, and that of patients diagnosed with breast cancer within the second year after delivery increased to 165. This number reached 222 within the third year after delivery. In the fourth year, the number of patients with breast cancer decreased to 147 (Fig 2). Among mothers who had delivered in 2010, the proportion of old age women (≥35 years) was higher among mothers with breast cancer than those without breast cancer. In addition, the proportion of primiparous women and women who underwent cesarean delivery were higher among mothers with breast cancer than among those without breast cancer (Table 3). For adjusted ORs, old age and cesarean delivery were associated with an increased risk of breast cancer (old age: OR, 2.777; 95% CI, 2.356–3.274 and cesarean delivery: OR, 1.211; 95% CI, 1.034–1.418), and primiparous status was associated with a decreased risk of breast cancer (OR, 0.673; 95% CI, 0.571–0.794; Table 4).

**Table 1:** Characteristics of Group 1 population who delivered during the 2010–2012 period with a total 1,384,551 deliveries.

	^a^Non-breast cancer (n = 1,383,656)	^b^Postpartum Breast cancer (n = 317)	*p*-value
**Median age (years)**	30.86	33.05	<0.0001^c^
**Old age (≥35 years)(%)**	241,750 (17.5)	102 (32.2)	<0.0001^d^
**Primipara**	709,559 (51.3)	129 (40.7)	0.0002^d^
**Pre-eclampsia (%)**	35,583 (2.6)	13 (4.1)	0.1129^d^
**Gestational diabetes (%)**	37,666 (2.7)	10 (3.2)	0.6444^d^
**Twin pregnancy**	20,901 (1.5)	5 (1.6)	0.9230^d^
**Cesarean delivery**	502,786 (36.3)	138 (43.5)	0.0085^d^

^a^The number of mothers who were not diagnosed with postpartum breast cancer within 365 days after childbirth during the 2010–2012 period was 1,383,656.

^b^The number of postpartum breast cancers who were diagnosed with breast cancer within 365 days after childbirth during the 2010–2012 period was 317.

^c^Student’s t-test was performed.

^d^Pearson chi-square test was performed.

**Table 2:** The adjusted ORs for the risk of postpartum breast cancer (diagnosed within 365 days after childbirth) in the Group 1 population with a total 1,384,551 deliveries during the 2010–2012 period.

	OR^a^ (95% CI)
**Old age (≥35 years)**	**2.003 (1.567–2.560)**
**Primipara**	**0.737 (0.585–0.928)**
**Pre-eclampsia**	**1.531 (0.874–2.680)**
**Gestational diabetes**	**0.978 (0.519–1.841)**
**Twin pregnancy**	**0.925 (0.379–2.257)**
**Cesarean delivery**	**1.237 (0.986–1.553)**

^a^Multivariate logistic regression analysis was performed. ORs were adjusted for all variables in the table.

OR, odds ratio; CI, confidence interval.

**Table 3:** Characteristics of Group 2 population who delivered in 2010 with a total of 457,924 deliveries (Group 2).

	^a^Non-breast cancer (n = 457,756)	^b^Postpartum breast cancer (n = 655)	*p*-value
**Median age (years)**	30.68	33.49	<0.0001^c^
**Old age (≥35 years)(%)**	75,759 (16.6)	254 (38.8)	<0.0001^d^
**Primipara**	232,475 (50.8)	237 (36.2)	<0.0001^d^
**Pre-eclampsia (%)**	11,497 (2.5)	19 (2.9)	0.5386^d^
**Gestational diabetes (%)**	9,924 (2.1)	24 (3.7)	0.0173^d^
**Twin pregnancy**	6,502 (1.4)	9 (1.4)	0.9163^d^
**Cesarean delivery**	164,432 (35.9)	286 (43.7)	<0.0001^d^

^a^Among the mothers who delivered in 2010, the number of mothers who were not subsequently diagnosed with breast cancer after childbirth during the 2010–2013 period was 457,756.

^b^Among the mothers who delivered in 2010, the number of patients who were subsequently diagnosed with breast cancer after childbirth during the 2010–2013 period was 655.

^c^Student’s t-test was performed.

^d^Pearson chi-square test was performed.

**Table 4:** The adjusted ORs for the risk of breast cancer (diagnosed within maximum 4 years after childbirth) in the Group 2 population with a total 457,924 deliveries in 2010.

	OR^a^ (95% CI)
**Old age (≥35 years)**	**2.777 (2.356–3.274)**
**Primipara**	**0.673 (0.571–0.794)**
**Pre-eclampsia**	**1.050 (0.663–1.663)**
**Gestational diabetes**	**1.368 (0.907–2.063)**
**Twin pregnancy**	**0.876 (0.451–1.700)**
**Cesarean delivery**	**1.211 (1.034–1.418)**

^a^Multivariate logistic regression analysis was performed. ORs were adjusted for all variables in the table.

OR, odds ratio; CI, confidence interval.

## Discussion

PABC is clinically significant because it affects younger population and has aggressive features [5,16–18]. In particular, postpartum breast cancer is more aggressive compared to breast cancer diagnosed during pregnancy [14], and the prognosis is poorer for postpartum breast cancer than for breast cancer diagnosed during pregnancy [5,14]. To date, the reason for poor prognosis of postpartum breast cancer is unknown. In this large, population-based study covering all Korean mothers, we identified the number of patients diagnosed with postpartum breast cancer based on the time from childbirth as well as the pregnancy-associated risk factors of postpartum breast cancer.

Within a year after childbirth, the number of postpartum breast cancer patients increased during the later period. More mothers were diagnosed with breast cancer during the 9–12 months after delivery than during the 0–3 and 4–6 months after delivery. According to the study on the rate of exclusive breast-feeding in Korean mothers using Korea National Health and Nutrition Examination Survey, the exclusive breast-feeding rate dropped sharply following the 6 months after childbirth, showing 60.9% at 1 month, 35.6% at 6 months, and 1.2% at 12 months after birth[19]. After mothers stopped breastfeeding, they could easily become aware of the changes in their breasts compared to during the lactation period owing to the stabilized features of the breast. In addition, their reluctance in undergoing diagnostic procedures could be eliminated after breastfeeding was stopped. Availability of self-awareness and detection abnormality of their breasts might be the important reason for the increased number of postpartum breast cancer patients in the later period within a year.

Additionally, the data of mothers who delivered in 2010 were traced until December 31, 2013. In the present analysis, while the number of patients diagnosed with postpartum breast cancer increased until the third year, this number slightly decreased in the fourth year. Previous studies reported that the timeframe within which the risk of breast cancer reaches its highest level is approximately 5 years after delivery [3,4]. Due to the relatively short follow-up duration, the shortest being 3 years in case of mothers who delivered on December 31, 2010, the number of patients diagnosed with breast cancer in the fourth year is likely to have been more than the recorded number. With a longer follow-up period, the risk of breast cancer according to the time elapsed from delivery could be precisely analyzed. However, we can safely conclude that the risk of breast cancer increased with time until the third year after delivery.

In the present study, age of ≥35 years and cesarean delivery were found to be significant risk factors of postpartum breast cancer. Recently, the incidence rates of breast cancer in young women have significantly increased worldwide [20,21]. Although the reasons for the increasing rate of breast cancer in young women is not clear, reproductive factors might be a possible reason. Since higher maternal age at first childbirth is associated with increased risk of PABC [1–4], recent trend in women to delay childbearing might lead to this increase of young age-onset breast cancer as well as PABC. Although we did not investigate the risk of age at the first childbirth of included women, women who give birth after 35 years of age should be aware of the risk of breast cancer because breast cancer is the second most common cancer type for this childbearing age group in Korea and the most common malignancy diagnosed postpartum [11,22].

Cesarean delivery was also identified as a significant risk factor for postpartum breast cancer in the present analysis. Little is known about the relationship between cesarean delivery and breast cancer. Kalan et al. reported in their analysis of 985 breast cancer patients that breast cancer in patients with a history of cesarean delivery had aggressive biological features [23]. The exact reason for this has not yet been elucidated; however, the authors suggested that lower exposure to oxytocin in patients who underwent cesarean delivery compared to patients who underwent vaginal delivery might influence the characteristics of the cancer. Oxytocin release increases during labor and lactation. However, oxytocin release decreases at cesarean delivery compared to vaginal delivery [24,25]. Moreover, oxytocin has shown anti-proliferative effects in several cancers as well as in breast cancer cells [26–28]. Mothers who underwent cesarean delivery are exposed to a low level of oxytocin because they do not undergo the labor process. In addition, mothers who underwent cesarean delivery were not prone to exclusive breast-feeding compared to mothers who underwent vaginal delivery [29,30]. Therefore, this might be a factor involved in the development of breast cancer. Lactation and oxytocin secretion are closely related. Several lactation-related factors associated with the risk of breast cancer that increases with reduced exposure to oxytocin include short lactation period, higher age at first breast feeding, and inadequate milk production [31]. We could not directly analyze the associations between lactation and risk of breast cancer because the insurance claim database did not have information on lactation; however, mothers who underwent cesarean delivery were expected to have insufficient lactation.

Although the exact mechanism of protective effect of oxytocin on breast cancer is not fully elucidated, low oxytocin exposure during gestation, delivery, or lactation is possibly associated with cancer development through several mechanisms [27]. Oxytocin modulates the expression and function of estrogen receptor alpha in human breast cancer cells and inhibits breast cancer cell proliferation by downregulating the mitogenic effects mediated by estrogens, important growth activators for breast cancer [32]. Cassoni et al. reported that oxytocin release blocks this mitogenic effect via the cAMP-PKA pathway, because cAMP levels increase upon the addition of oxytocin, while PKA inhibitors block the anti-mitogenic effect of oxytocin in breast cancer cell lines [26]. In addition, oxytocin stimulates the myoepithelial cells of the breast, which play a role in breast morphogenesis and tumor suppression, and thus lead to the elimination of potential carcinogenic substances accumulated in the ductal system [33,34]. Furthermore, oxytocin showed protective roles against immunologic damages [35]. Decreased anti-inflammatory response owing to lesser release of oxytocin might promote the development of cancer. A decrease in the immune response leads to the recruitment of fewer anti-tumor immune cells; consequently, decreased anti-tumor immune response might promote cancer development. Moreover, postpartum breast tissue undergoes changes that make it more prone to cancer development. These changes include the involution of the postpartum mammary gland characterized by the elimination of secretory lobules laid down in preparation for lactation, massive epithelial cell death, stromal remodeling, and immune cell infiltration [36]. In the study of Martinson et al., the authors focused on breast tissue remodeling that resembled a wound-healing–like immune program; this could lead to the progression and aggressive behavior of the breast cancer [37]. In addition, Lyons et al. reported that postpartum mammary gland tissue acquired enhanced prolymphangiogenic activity, because physiologic cyclooxygenase-2–dependent lymphangiogenesis during involution might contribute to peritumoral lymphatic expansion, increased tumor size, invasion, and distant metastases [38]. Therefore, changes in the breast microenvironment during postpartum period along with the effect of low oxytocin release might particularly increase the risk of breast cancer after delivery.

Parity is reported to show opposite effects in young age-onset and old age-onset breast cancer. A higher number of childbirths is a well-known protective factor against postmenopausal breast cancer [39]. However, nulliparity was found to be associated with a decreased risk of young age-onset breast cancer in some studies [40–43]. In a case-control study from Poland, nulliparity reduced breast cancer risk among ages 25–39 years while it increased the relative risk in women of ages 40–74 years [42]. In this present study, in which all included women had had at least one delivery, similar results were observed. Here, primiparous women showed a lower risk of breast cancer compared to other multiparous women. Since we could identify only the primiparous women with special medical claim, but the number of parity of the included women, we could only investigate the risk of primiparous women. Therefore, we cannot assert that lower parity is a protective risk factor against postpartum breast cancer; however, we can suggest that at least primiparous status is associated with a lower risk for postpartum breast cancer, and childbirths might increase the risk of postpartum breast cancer, particularly young age-onset breast cancer. This coincides with other previous reports in terms that fewer experience of childbirth is more protective against young age-onset breast cancer while repetitive experiences to pregnancy is not protective against young age-onset breast cancer. The reason for this is unclear; however, the hormonal effects in repetitive pregnancies might influence differently between young age-onset breast cancer and old age-onset breast cancer. Further investigations are warranted.

The present study had several limitations. First, individual cancer characteristics could not be obtained because this study was based on the insurance claim data from the Korea National Health Insurance Claims Database of the HIRA, a database designed for cost claim issues, not for research. In addition, we could not analyze patient characteristics such as age at menarche, body mass index, family history of breast cancer, lactation, oral contraceptive use, and alcohol consumption in detail. Moreover, we could not investigate the characteristics of labor such as the cause and type of cesarean delivery, and whether planned or following labor. Nevertheless, this is the first study including all maternities in Korea over a period of 3 years. In addition, the cancer patients included in the present study were tightly followed up by the hospital because they were registered and supported by the government through the critically ill patients benefit program of Korea. Therefore, the number of patients and extracted data are reliable.

The special clinical or biological characteristics of PABC have been identified after extensive investigations. Earlier diagnosis is an important determinant of prognosis in patients with PABC that have worse prognoses than patients with non-PABC. Therefore, breast cancer should be considered in patients having the risk factors analyzed in this study. Careful self-examination, physician’s caution, and active examination are required for early diagnosis. Moreover, diagnostic tools having higher sensitivity and specificity compared to the existing ones should be developed for patients in this condition. Subsequently, special screening plans should be designed for mothers who visit the hospital frequently owing to pregnancy and delivery.

In conclusion, old age (≥35 years), cesarean delivery are significant risk factors for postpartum breast cancer, and primiparous women have a lower risk of developing postpartum breast cancer. Mothers and physicians should exercise caution concerning breast cancer especially in the presence of these risk factors.
